# Gait profile score and movement analysis profile in patients with Parkinson's disease during concurrent cognitive load

**DOI:** 10.1590/bjpt-rbf.2014.0049

**Published:** 2014

**Authors:** Danielli S. Speciali, Elaine M. Oliveira, Jefferson R. Cardoso, João C. F. Correa, Richard Baker, Paulo R. G. Lucareli

**Affiliations:** 1 Laboratório de Estudos do Movimento do Hospital Israelita Albert Einstein (LEME), São Paulo, SP, Brazil; 2 Centro Universitário das Faculdades Metropolitanas Unidas (FMU), São Paulo, SP, Brazil; 3 Associação de Assistência à Criança Deficiente (AACD), São Paulo, SP, Brazil; 4 Northeasertern University, Boston, MA, USA; 5 Laboratório de Biomecânica, Universidade Estadual de Londrina (UEL), Londrina, PR, Brazil; 6 Programa de Pós-Graduação em Ciências da Reabilitação, Universidade Nove de Julho (UNINOVE), São Paulo, SP, Brazil; 7 Salford Gait Analysis Service, University of Salford, Salford, United Kingdom

**Keywords:** Parkinson's disease, gait, kinematics, attention, rehabilitation

## Abstract

**Background:**

Gait disorders are common in individuals with Parkinson's Disease (PD) and the concurrent performance of motor and cognitive tasks can have marked effects on gait. The Gait Profile Score (GPS) and the Movement Analysis Profile (MAP) were developed in order to summarize the data of kinematics and facilitate understanding of the results of gait analysis.

**Objective:**

To investigate the effectiveness of the GPS and MAP in the quantification of changes in gait during a concurrent cognitive load while walking in adults with and without PD.

**Method:**

Fourteen patients with idiopathic PD and nine healthy subjects participated in the study. All subjects performed single and dual walking tasks. The GPS/MAP was computed from three-dimensional gait analysis data.

**Results:**

Differences were found between tasks for GPS (P<0.05) and Gait Variable Score (GVS) (pelvic rotation, knee flexion-extension and ankle dorsiflexion-plantarflexion) (P<0.05) in the PD group. An interaction between task and group was observed for GPS (P<0.01) for the right side (Cohen's ¯d=0.99), left side (Cohen's ¯d=0.91), and overall (Cohen's ¯d=0.88). No interaction was observed only for hip internal-external rotation and foot internal-external progression GVS variables in the PD group.

**Conclusions:**

The results showed gait impairment during the dual task and suggest that GPS/MAP may be used to evaluate the effects of concurrent cognitive load while walking in patients with PD.

## Introduction

Walking is one of the tasks most affected by idiopathic Parkinson's disease (PD). A particular problem is the way that the condition interferes with the management of attention to stimuli when two tasks are performed simultaneously^1^. In daily living, the environment invariably forces an individual to divide his or her attention among various stimuli that occur simultaneously and often require motor responses. The ability to perform such concurrent tasks is particularly limited in patients with PD, especially when one of the tasks is walking. This leads to the impairment of one or both tasks, with a negative impact on the activities of daily life^2^. The potential consequences of gait impairment in PD are significant and include increased disability, a greater risk of falls, and a reduced quality of life^3^.

Defective functioning of the basal ganglia results in increased cortical involvement in motor control among individuals with PD, leading to an increase in difficulty managing dual tasks^4^. Moreover, the ability to prioritize gait and balance appropriately during dual-task activities is impaired in patients with this disease, likely due to the deterioration of executive processes, which is correlated with increased gait variability^1^. Individuals with PD exhibit an increase in gait variability in response to dual tasks, which places increased demands on attention resources^4^ ^-^ ^7^.

The relationship between cognitive function and gait impairment has received considerable attention in recent years. Biomechanical studies have addressed spatiotemporal gait parameters in PD^8^ ^-^ ^10^, but few have focused on angular parameters. A reduction in the angular excursion of lower limb joints has been noted in parkinsonian syndromes with the primary gait deficit in PD having been described as an inability to generate sufficient range of motion^11^ ^-^ ^13^.

Three-dimensional gait analysis (3DGA) measures angular changes in lower limb joints during locomotion. Typically, kinematic graphs are generated to assess gait quality, to guide decisions regarding the management of gait disorders, and to help evaluate treatment outcomes. Although routinely viewed, kinematic graphs are complex and require significant expertise to interpret and describe^14^. Due to the large amount of information generated by gait analysis, a number of indices and scores have been designed to condense complex kinematic data and provide simple, easy-to-interpret data for use in clinical practice^15^.

The Gait Profile Score (GPS) was developed to summarize data on kinematics and to facilitate the understanding of the results of gait analysis. The GPS can be broken down to provide the Gait Variable Score (GVS), based on nine kinematic variables^16^ and establish a Movement Analysis Profile (MAP), which describes the magnitude of the deviation of those nine variables across the gait cycle^17^ ^-^ ^19^.

To our knowledge, no studies have previously employed the GPS to evaluate the effects of a dual task (concurrent cognitive load while walking) on adults with PD.

Thus, the aim of the present study was to investigate the effectiveness of the GPS and the MAP regarding the quantification of changes in gait during a dual task performed by healthy adults and individuals with Parkinson's disease.

## Method

### Participants

From a total of 14 individuals diagnosed with idiopathic PD, 7 female and 7 male participated in the present study [mean age and standard deviation (SD): 67.5 years (5.6)]. The following were the inclusion criteria for the PD group (PDG): ability to walk barefoot independently without a gait-assistance device; absence of any other neurologic disorder or dementia, having achieved a score of ≥24 on the Mini-Mental State Examination^20^; classification Stages 2 and 3 on the Modified Hoehn and Yahr Scale^21^; and in the "ON" phase of the active medication cycle. The Freezing of Gait questionnaire (FOG-Q)^22^ also was used. Thirty individuals were excluded due to the following exclusion criteria: subjects with other types of PD, individuals with rheumatic disease, and orthopaedic problems or previous orthopaedic surgery of the lower limbs.

The control group (CG) consisted of nine healthy elderly individuals (5 female and 4 male) with a mean age of 65.1 years (SD: 5.3) with no history of pre-existing diseases or complaints affecting activities of daily living, specifically gait; having achieved a score of ≥24 on the Mini-Mental State Examination.

All patients participated in the same physical therapy program once a week. The healthy elderly did not perform physical activity. All subjects gave informed consent to perform the experimental procedure and the study received approval from the local ethics committee Centro Universitário São Camilo, São Paulo, SP, Brazil (protocol 93/08).

### Procedures

The participants were informed regarding the data acquisition procedures, familiarized with the place at which data would be collected and trained so that gait would be as normal as possible. The participants did not use any gait-assistance devices and absolute silence in the laboratory was requested during data acquisition so that no noises interfered with the participant's attention during the tasks. The assessments were done at the same time period and on the same day.

Initially, the subjects walked barefoot at a comfortable speed with no other competing tasks (simple task) and then rested for 20 minutes. A dual task was then implemented, requiring the participants' attention to an activity during gait. The dual task involved walking while doing a cognitive task which consisted of a mathematical test of decreasing consecutive subtraction. The participants walked while performing a set of seven subtractions out loud, starting from 500^11^. No instructions were given regarding the priority of one task over the other (walking vs. cognitive task). All were instructed to walk on a track which was 1.5 meters wide × 6.0 meters long.

### Equipment

An eight-camera motion analysis system (Motion Analysis Corporation, Santa Rosa, CA, USA) (sample rate, 60 Hz and fourth-order Butterworth filter with cut-off frequency of 8 Hz) was used to capture the three-dimensional marker trajectories. A total of 23 reflective markers were attached to the skin of each participant at specific anatomic points based on the Helen Hayes model^23^. The markers were placed on the iliac spine, thighs, lateral femoral epicondyle, legs, lateral malleolus, metatarsals, calcaneus and hallux.

### Data processing and analysis

Kinematic variables for analysis were based on the Helen Hayes biomechanical model used in the Orthotrack^(r)^ 6.2 software (Motion Analysis Corporation, Santa Rosa, CA, USA). All data obtained from the 3DGA were normalized to a percentage of the gait cycle and the angular gait values were exported as ASCII archives from the Orthotrack^(r)^ program to Microsoft Excel^(r)^ for each group (Parkinson's disease and control) under the simple task and dual task conditions. A total of six gait cycles were used to obtain these values.

Subsequently, the GPS scores for the PD and control groups were calculated for each leg in relation to data for normal healthy adults captured at the movement analysis laboratory. The GPS was based upon 15 clinically important kinematic variables (pelvic tilt, obliquity, rotation from one side and hip flexion, abduction, internal rotation, knee flexion, dorsiflexion and foot progression for left and right sides)^24^. The GPS represented the root mean square difference between a particular gait trial and averaged data from individuals without a gait impairment^19^ ^,^ ^25^. Neither the GPS nor the MAP components were normally distributed; thus, logarithmic transformations were performed before applying parametric statistics to the data.

Analysis of variance (ANOVA) was used for comparisons between groups. For the overall GPS and pelvic tilt, obliquity and rotation, a two-way ANOVA was used considering group and task as the factors. For the other variables, a three-way ANOVA was used considering side, group and task as the factors, after checking the assumptions of the equality in error variances (Levene). Interactions between variables were also analyzed. The existence of an interaction may indicate, for example, whether differences between groups only occurred on a particular side. If the F test was significant, multiple comparisons were performed using the Bonferroni test. Cohen's ^-^ *d *was used to measure the effect size for both the CG (normal vs dual task) and PDG (normal vs dual task) for power analysis purposes^26^. The effect size was classified as high, medium or low. Statistical significance in all tests was 5% (*P*<0.05). The Statistical Package for Social Sciences, version 15, was used for the analysis (SPSS Inc., Chicago, USA).

## Results

Table 1 displays the descriptive and demographic characteristics at baseline for the control and PD groups. Table 2 summarizes the results in mean and standard deviation values for all variables during normal gait and gait with dual task for both groups.

**Table 1:** Clinical and demographic characteristics of patients in the Parkinson's disease group (PDG; n=14) and control group (CG; n=9) of healthy individuals.

	CG	PDG
Age (years)	65.11 (5.3)	67.50 (5.6)
Male/Female	4M/5F	7M/7F
Height (m)	1.64 (0.05)	1.66 (0.10)
Body Mass (kg)	68.11 (10.52)	68.50 (15.16)
*Gait velocity (m/s)	1.01 (1.48)	0.95 (0.26)
Mini-Mental State Examination	28.11 (2.08)	27.64 (1.9)
Modified Hoehn & Yahr stage – (in each stage)	-	2 (4); 2.5 (8); 3(2)
Freezing of gait questionnaire	-	10.7 (6.23)
Medication (number of patients)	-	Levodopa (14) / Carbidopa (14) / Entacapone (2) / Bromocriptine (1)

Values expressed in mean (standard deviation); *During normal gait; (-) data not collected.

**Table 2:** GPS/MAP during normal gait and gait with task on both sides in control group (CG) and Parkinson's disease group (PDG).

		Normal Gait	Dual Task	Effect size Group vs Task
**GPS_Overall ** ^**a**^^†^^**,b**^^†^^**,d**^^§^	CG	6.65 (1.28)	7.09 (1.15)	-
	PDG	9.17 (1.18)	10.30 (1.37)	0.88
**Pelvic_ant_pst ** ^**a**^^†^^**,b**^^§^^**,d**^^§^	CG	5.13 (2.27)	5.25 (2.60)	-
	PDG	5.63 (1.93)	6.87 (1.64)	0.69
**Pelvic_obliquity ** ^**a**^^†^^**,b**^^§^^**,d**^^§^	CG	2.73 (1.09)	2.79 (1.11)	-
	PDG	2.87 (0.98)	3.12 (0.82)	0.30
**Pelvic_rotation ** ^**a**^^†^^**,b**^^†^^**,d**^^§^	CG	3.44 (1.53)	3.83 (0.92)	-
	PDG	4.57 (1.44)	5.98 (2.88)	0.61
		** Right**			** Left**		
		** Normal Gait**	** Dual Task **	** Effect size Group vs Task**	** Normal Gait**	** Dual Task**	** Effect size Group vs Task**
**GPS ** ^**a**^^†^^**,b**^^†^^**,d**^^§^	CG	6.25 (1.54)	6.56 (1.31)	-	6.18 (1.16)	6.62 (1.23)	-
	PDG	8.08 (1.61)	9.69 (1.64)	0.99	8.04 (1.21)	9.22 (1.36)	0.91
**Hip_flex_ext ** ^**a**^^†^^**,b**^^§^^**,d**^^§^	CG	7.22 (1.84)	7.43 (1.62)	-	6.60 (1.75)	6.90 (1.56)	-
	PDG	10.62 (5.36)	12.13 (4.73)	0.30	10.18 (4.01)	11.87 (4.09)	0.41
**Hip_ad_abd ** ^**b**^^§^^**,d**^^§^	CG	5.27 (2.16)	5.66 (2.39)	-	4.96 (1.86)	4.74 (1.72)	-
	PDG	4.64 (2.00)	5.98 (2.64)	0.57	5.03 (2.13)	5.80 (2.83)	0.30
**Hip_int_ext ** ^**a**^^†^	CG	5.21 (2.25)	5.08 (0.98)	-	4.87 (1.14)	5.62 (0.91)	-
	PDG	10.46 (3.45)	10.70 (3.10)		10.71 (2.92)	10.71 (3.36)	
**Knee_flex_ext ** ^**a**^^†^^**,b**^^†^^**,d**^^§^	CG	6.55 (2.02)	7.28 (2.04)	-	6.46 (1.87)	7.54 (1.81)	-
	PDG	12.73 (4.25)	15.09 (4.10)	0.56	12.76 (3.46)	14.73 (3.59)	0.55
**Ankle_Dor_plan ** ^**a**^^†^^**,b**^^†^^**,d**^^†^	CG	4.87 (1.14)	5.35 (1.20)	-	4.53 (1.87)	5.22 (1.64)	-
	PDG	8.08 (2.19)	10.04 (2.17)	0.89	8.32 (2.61)	10.37 (2.55)	0.80
**Foot_int_ext**	CG	7.86 (5.05)	8.22 (3.69)	-	6.36 (3.02)	7.96 (3.59)	-
	PDG	8.22 (3.69)	8.68 (6.21)		6.90 (3.50)	9.38 (4.92)	

a Mean difference between groups

b Mean difference between task

c Mean difference between side, dinterference effect between group and task, einterference effect between group and side, finterference effect between task and side, ginterference effect between group, task and side

† Mean difference is significant at the .050 level

§ Mean difference is significant at the .001 level

**Ant_post:** = anteversion_retroversion

**flex_ext:** = flexion_extension

**ad_abd:** = adduction_abduction

**dor_plan:** = dorsiflexion_plantarflexion

**int_ext:** = internal_external rotation.

Statistically significant differences were found between groups for GPS and GVS variables (pelvic tilt, pelvic obliquity, pelvic rotation, hip flexion-extension, hip internal-external rotation, knee flexion-extension and ankle dorsiflexion-plantar flexion). Differences were found between tasks regarding the GPS and GVS (tilt pelvic, pelvic obliquity, pelvic rotation, hip flexion-extension, hip adduction-abduction, knee flexion-extension and ankle dorsiflexion-plantar flexion) in PDG. When sides were compared, differences were not found (Table 2).

An interaction between task and group was observed in GPS and almost all GVS variables, except for hip internal-external rotation and foot internal-external rotation in PDG. No interactions between side and task or side, task and group were observed. The effect size observed between the PD group and task interaction was high for GPS: right side (Cohen's ¯d=0.99), left side (Cohen's ¯d=0.91) and overall (Cohen's ¯d=0.88). The effect size for GVS was medium in all variables (Table 2).

## Discussion

The aim of the present study was to investigate the effectiveness of the GPS/MAP component regarding the quantification of changes in gait during dual tasking in individuals with PD. Previous studies report strong, significant correlations between the GPS/MAP component scores and kinematic gait deviation^19^ ^,^ ^27^. However, no studies have employed the GPS/MAP to assess the gait of individuals with PD during a dual-task activity. The representation of angular kinematics through this score may be useful in interpreting the results of analyses of the main changes in gait in this population.

There is a growing line of evidence showing that concurrent cognitive load while walking has significant ramifications on the gait of patients with PD. Consistent with previous studies, the results of the present investigation demonstrated that dual tasking and attention influence gait^5^ ^,^ ^10^ ^,^ ^12^.

**Figure 1:**
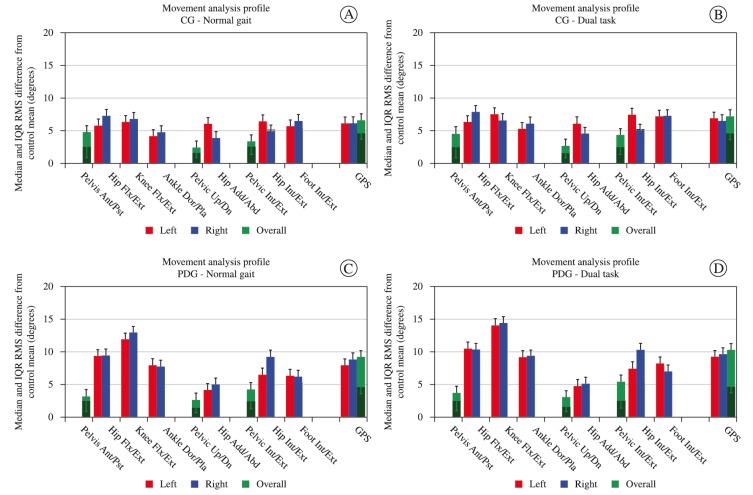
Gait profile score and movement analysis profile in control and Parkinson's disease groups during normal and concurrent cognitive load. A = CG during Normal Gait; B = CG during Dual Task; C= PDG during Normal Gait; D = PDG during Dual Task. Ant_post = anteversion_retroversion; flex_ext = flexion_extension; ad_abd = adduction_abduction; dor_plan = dorsiflexion_plantarflexion; int_ext = internal_external rotation.

The PDG exhibited different movement patterns when compared to healthy individuals, as demonstrated by a visual comparison of the MAP in Figure 1 (A/B and C/D). When the cognitive task was added, the PDG changed the gait pattern. These findings are seen in the results of the GVS (pelvic tilt, pelvic obliquity, pelvic rotation, hip flexion-extension, hip adduction-abduction, knee flexion-extension and ankle dorsiflexion-plantar flexion) and, consequently, in the GPS. The analysis of interactions between factors revealed that the GPS and GVS variables were only different for the PD group during the dual task. These results are supported by those obtained from previous studies on the effect of the dual task on gait in patients with PD, which report changes in the kinematics of the gait pattern^1^ ^,^ ^4^ ^,^ ^28^ ^-^ ^30^.

The PDG showed significant differences during gait with dual task. Gait alterations in patients with PD and elderly individuals submitted to dual-task activities have been described in the literature, but no previous study has employed the GPS/MAP. The MAP provided an overview of the gait deviation from the normal pattern, illustrating changes due to interference from the dual task. Gait in patients with PD is characterized by a decrease in the angular range^12^. Previous studies have shown that the range of motion of the knee and ankle joint in the sagittal plane undergoes significant variation during the gait cycle, with a reduction in knee and ankle range of motion during a dual task^12^ ^,^ ^13^. Some authors report that, among patients with PD on levodopa, dual tasks lead to a significant increase in multi-joint and multi-plane lower limb joint range of motion^11^ ^,^ ^12^.

Gait deficits are exacerbated during the performance of a dual task by patients with PD, as the need to concentrate on both walking and a concurrent task exceeds the available attention resources^10^. In PD, the extra attention needed to perform the task or hyperstimulation provoked by unexpected stimuli induces a hypo-excitability that can be manifested as a motor block. However, during simultaneous tasks, the response time to the cognitive task was reduced due to the increase in attention needed to perform the motor task, which resulted in the exacerbation of gait defects during the performance of a dual task exercise among patients with PD^4^ ^,^ ^29^.

Our findings show an increase in the GPS scores (sides and overall) with a high effect size, which means that, in general, the gait pattern changed during a concurrent cognitive load. Based on the effect size, the increase in the GVS scores showed that ankle dorsiflexion-plantar flexion, and pelvic anteversion and rotation were more affected with a high effect size and knee flexion-extension; hip flexion-extension, adduction-abduction and pelvic obliquity with a medium effect size in the PDG, suggesting that the dual task exerted substantial influence on balance strategies, and might be related to the risk of falls in these individuals.

Differences were found between tasks for GPS and GVS in the PD group. Studies reported that when two tasks requiring a high degree of information processing were performed simultaneously, the performance of one or both was diminished. This impairment in the primary task and/or secondary task resulted from the fact that the two tasks competed for similar processing demands^4^ ^,^ ^10^. Dual tasking has also been used to identify the risk of falls in patients with PD due to the secondary relationship to postural strategies stemming from the loss of attention and a reduction in gait performance during a dual task^10^ ^,^ ^12^. The mathematical problems introduced during gait lead to a high degree of competition for executive motor function, suggesting that the automaticity of the performance under the complex conditions of walking is multidimensional^29^ ^-^ ^31^.

There are few reports of the use of GPS/MAP in clinical research. Some authors observed a strong linear correlation between the GPS and scales of physical function in patients with cerebral palsy. Changes in GPS of 1.6º represents a uniform change of just 1.6º across all gait parameters and represents a mix of much larger changes in some of the constituents of the MAP with much smaller changes in others. Similar factors apply across the gait cycle with substantial changes at critical phases within the gait cycle often being balanced by more modest changes at others. A minimally clinically important difference of 1.6º seems appropriate for the individual GVS in patients with cerebral palsy^24^. However, no studies about the minimal clinically important difference of GPS/MAP for patients with PD were found. There are descriptions only for individuals with cerebral palsy, which differs greatly from the study population, make it impossible to establish any correlation.

The results of the present study have important implications for the rehabilitation of individuals with motor impairment associated with PD and demonstrate that the use of dual tasks should be included in rehabilitation processes. Thus, MAP can be used to complement the traditional presentation of gait kinematics. Although individual terms are selected (unlike other indexes in the literature), the GPS/MAP score points to the gait in general terms and should not be used separately to interpret the origin of changes in gait pattern.

The GPS/MAP may provide a summary of gait data that indicates asymmetry and the relative magnitude of deviations from each of the typical kinematic variables. As clinical decision making requires inspection of individual joint kinematics, we suggest that the GPS scores may reflect the clinical judgment more closely than an overall gait index. Despite the lack of studies, the use of GPS/MAP in patients with PD during a cognitive task showed a sensitive tool to point out the main gait differences in this population, providing simple and easy interpretation for clinical practice measures.

Limitations of this study include its relatively small sample size and the intrinsic procedural limits of 3DG. To minimize this, the effect size (Cohen's ^-^ *d*) was presented, which varied from 0.30 to 0.99, representing values for the PDG normal gait from the 62th to the 84th percentile of the PDG dual task (from medium to large effect size). Further studies are needed to understanding this complex relationship, which has implications for the rehabilitation of gait among patients with PD.
